# Vascular smooth muscle cell activation depends on NOS-1-derived NO and consequent peroxynitrite generation

**DOI:** 10.1186/cc12981

**Published:** 2013-11-05

**Authors:** Karin Scheschowitsch, Regina de Sordi, João Alfredo de Moraes, Christina Barja-Fidalgo, Jamil Assreuy

**Affiliations:** 1 Department of Pharmacology, UFSC, Florianópolis, SC, Brazil; 2 Department of Pharmacology, UERJ, Rio de Janeiro, RJ, Brazil

## Background

Low levels of nitric oxide (NO) play a key role in vascular tonus maintenance. Previous results from our laboratory show that hypotension and mortality during sepsis are prevented by the early administration of NOS-1 inhibitors. The aim of this study was thus to investigate the role of NOS-1 and NOS-3-derived NO and of other reactive oxygen species (ROS) in smooth muscle cell activation.

## Materials and methods

Smooth muscle cell line of rat aorta (A7r5) was used. Control cells and NOS-1 or NOS-3 silenced cells (siNOS-1 and siNOS-3, respectively) were stimulated with LPS 1 µg/ml and IFN 200 U/ml (LPS/IFN). NO and ROS production was assessed with fluorescent probes. NOS content was evaluated by western blot and NOS-2 activity was indirectly measured by Griess reaction. Further, control cells were treated for 30 minutes with a NO scavenger (c-PTIO), a NOS inhibitor (7-NI) or a NADPH oxidase inhibitor (DPI) before stimulation. Immunofluorescence was used to evaluate protein nitration and NF-κB nuclear translocation. To confirm the role of peroxynitrite in cell activation, control cells were stimulated with a sub-effective amount of LPS/IFN together with a NO donor and a superoxide anion generator and treated with a NOS-2 inhibitor 4 hours after stimulation. Griess reaction was performed 48 hours after. Statistical comparisons were performed by two-way ANOVA followed by the Bonferroni test.

## Results

A7r5 control cells stimulated with LPS/IFN presented a rapid increase in intracellular NO and ROS content. These increases were prevented by c-PTIO, 7-NI and DPI, as well as in siNOS-1 and siNOS-3 cells. NOS-2 was only expressed after cell stimulation. Control cells incubated with c-PTIO or 7-NI and stimulated with LPS/IFN presented a diminished NOS-2 expression and activity. Only in siNOS-1 cells was NOS-2 expression and activity also reduced. Nuclear translocation of NF-κB and positive nitrotyrosine reaction were reduced in c-PTIO or 7-NI treated groups. Sub-effective concentrations of LPS/IFN did not induce significant nitrite production. However, when sub-effective LPS/IFN was associated with the production of low concentrations of peroxynitrite, nitrite accumulation was as high as in cells stimulated with activating concentrations of LPS/IFN.

## Conclusions

We show for the first time the importance of NOS-1-derived NO and peroxynitrite for smooth muscle cell activation. Cell stimulation with LPS/IFN causes an early NOS-1-derived NO pulse and a ROS pulse that forms peroxynitrite. The interplay between these species seems to be key events for NF-κB nuclear translocation and NOS-2 expression.

